# Eugenol Polysiloxane-Polycarbonate/Graphene Nanocomposite: Enhanced in Thermostability and Barrier Property

**DOI:** 10.3390/nano9121747

**Published:** 2019-12-09

**Authors:** Xiaoyan Pang, Mingde Chen, Junwei Fu, Zehua Lin, Yuming Li, Jianxin Wu, Jie Yan, Xunjun Chen, Jianfang Ge

**Affiliations:** College of Chemistry and Chemical Engineering, Zhongkai University of Agriculture and Engineering, Guangzhou 510225, China; shelly_pxy@163.com (X.P.); mingde_chen09@163.com (M.C.); a1399426303@163.com (J.F.); wlxzhh@163.com (Z.L.); 17820165675@163.com (Y.L.); wu869887924@163.com (J.W.); yanjie0001@126.com (J.Y.); chenxj@zhku.edu.cn (X.C.)

**Keywords:** eugenol polysiloxane-polycarbonate, graphene, thermostability, barrier property

## Abstract

Graphene (GR) was used to blend with eugenol polysiloxane-polycarbonate (Si-PC) copolymer to prepare a Si-PC/GR nanocomposite via a solution blending method and the impact of graphene on the properties of Si-PC/GR nanocomposite was investigated. The morphology and structure of the Si-PC/GR nanocomposite were characterized. Combining morphology and property analysis, the result showed that when the graphene dispersed uniformly in the Si-PC matrix, the mechanical properties, thermostability and barrier property of Si-PC/GR nanocomposite were enhanced. Compared with Si-PC copolymer, the pyrolytic temperature of Si-PC/2.5%GR nanocomposite at 5% weight loss was 434.3 °C, which was 20.6 °C higher than Si-PC copolymer; and the oxygen barrier value of Si-PC/1.5%GR nanocomposite decreased to 160.2 cm^3^/m^2^ 24 h 0.1 MPa, which was 53.2 less than pure Si-PC. The mechanical properties of Si-PC/GR nanocomposite were enhanced with an appropriate additive amount of graphene. The hydrophobicity also had been enhanced at the meantime.

## 1. Introduction

As a kind of engineering thermoplastic, aromatic polycarbonate (PC) possesses excellent properties. It is used in glass, machine parts and plane cabin covers [1,2]. PC, with a high melt viscosity, is hard to machine and the product is likely to stress crack, which limits its further application, so modifications of PC have attracted many researchers [3]. With a soft Si–O–Si main chain, polysiloxane exhibits high and low temperature resistance, weather resistance, hydrophobicity and low-viscosity, and is usually used to modify plastic and rubber [4,5]. Eugenol polysiloxane, a new type of polysiloxane with two phenolic hydroxyl terminal groups, was synthesized via reaction between eugenol and hydrogenterminated polysiloxane, as our previous research reported [6]. Eugenol is a common bio-phenol in the chemical synthesis, and eugenol polysiloxane was used in the polymer modification for its chemical reactivity, antibacterial property and benzene ring which could improve the rigidity of polymer [7,8,9,10,11]. Eugenol pysiloxane-polycarbonate (Si-PC) copolymer was synthesized via copolymerization between eugenol polysiloxane and PC monomer, and thusm reduced the melt viscosity, improved the toughness and improved the thermal properties of PC [12,13].

The functional fillers could enhance the thermostability and mechanical and barrier properties of composite, and thus meet application requirements in special fields [14,15,16]. Yoonessi et al. [17] prepared carbon nanotube epoxy nanocomposite and studied the effect of interfacial modifications on the dynamic mechanical properties of the nanocomposite. Graphene, with high stiffness and extraordinary thermal stability and barrier property, was used to be filler to improve the performance of composites [18,19,20]. Yoonessi [21] prepared graphene/polycarbonate nanocomposite via two different blending methods and the result showed that the electrical property and dynamic tensile moduli had been enhanced with increasing the additive amount of graphene nanosheets.

In this study, graphene, without further modifying, was used to blend with Si-PC to prepare Si-PC/GR nanocomposite via a solution blending method and the additive amount of graphene was in the range of 0.5–2.5 wt%. Graphene was expected to improve the mechanical properties, thermostability and barrier property of nanocomposite. This research studied the impact of additive amounts of graphene on the properties of Si-PC/GR nanocomposite. The chemical structure and morphology of the Si-PC/GR nanocomposite were analyzed. The thermostability, hydrophobicity, barrier property and mechanical properties of the Si-PC/GR nanocomposite were tested.

## 2. Materials and Methods

### 2.1. Materials

Si-PC (Mw = 30,314 g/mol) was synthesized in our laboratory and the mass fraction of eugenol polysiloxane was 10 wt% [13]. Graphene nanoparticle was purchased from Changzhou Sixth Element Material Technology Corporation and the specific surface area of graphene nanoparticle was 160–240 m^2^/g and the particle size was less than 10 um. Dichloromethane was obtained from Tianjianshi Baishi Chemical Corporation.

### 2.2. Preparation of Si-PC/GR Nanocomposite

**Scheme 1:**
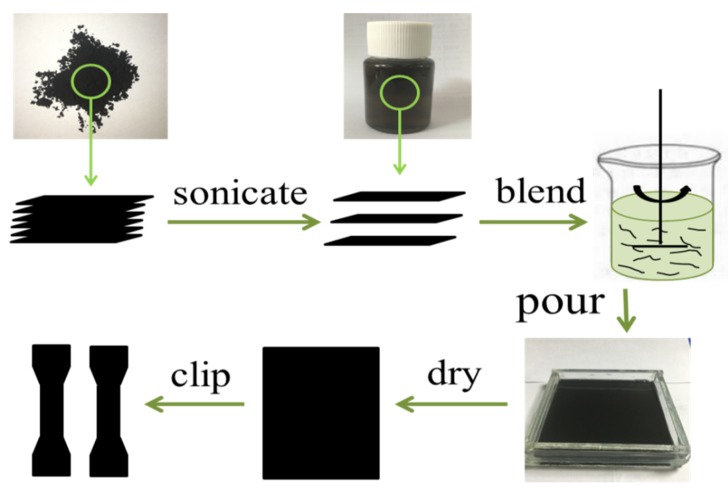
Preparation of Si-PC/GR nanocomposite.

The Si-PC/GR nanocomposites were fabricated via a solution blending method. Graphene nanoparticles were added into the anhydrous dichloromethane. A dilute graphene nanoparticle dichloromethane dispersion (5 × 10^−4^ g/mL) was sonicated for 1 h and then mixed with Si-PC dichloromethane solution (0.1 g/mL). The mixture was sonicated for 1 h at room temperature. The mixture solution was poured into the mold and evaporated at ambient temperature for 8 h. The Si-PC/GR nanocomposites were obtained (0.5–2.5 wt% of graphene) after dichloromethane removal. Scheme 1 shows the preparation of Si-PC/GR nanocomposite.

### 2.3. Chemical Structure of Si-PC/GR Nanocomposite

Si-PC/GR nanocomposite membranes were dried in the vacuum drying oven at 80 °C for 4 h, and then a Fourier transform infrared (FTIR) spectrum of Si-PC/GR nanocomposite membrane was conducted on a FTIR spectrometer 100 (Perkin-Elmer Corporation, Fremont, CA, USA) in an atmospheric environment via attenuated total reflectance method (ATR). The scanning wave number was at the range of 400–4000 cm^−1^.

### 2.4. Morphology of Si-PC/GR Nanocomposite

The thin Si-PC/GR nanocomposite membrane was fractured in liquid nitrogen and then dried. The fracture surface was observed by scanning electron microscopy (SEM, EVO 18, Carl Zeiss, Jena, Germany) after coating with gold for 45 s.

### 2.5. TEM Characterization of Graphene

A transmission electron microscope (TEM, JEM 100CX, Leica, Wetzlar, Germany) took images of graphene. The graphene layers were sonicated for 30 min to obtain dilute graphene nanoparticle dispersion. The graphene nanoparticle was dissolved in tetrahydrofuran.

### 2.6. Thermostability

The thermal decomposition curve of Si-PC/GR nanocomposite was tested on a TG/DTA thermal analyzer (Mettler-Toledo AG Corporation, Columbus, OH, USA) to measure the temperature and rate of thermal decomposition. The temperature was set to the range of 40–700 °C and the heating rate was 10 °C/min under nitrogen atmosphere.

### 2.7. Mechanical Properties

The tensile strength and nominal fracture strain of Si-PC/GR nanocomposites membranes were measured on a tensile machine (CMT6503, Shenzhen MTS Test Machine Company Ltd., Shenzhen, China). According to GB/T1040.3-2006-B1standard, the membranes were clipped into standard shapes and the sensor used was 100 N. The average value was calculated after three tests.

### 2.8. Oxygen Barrier Property

According to the GB/T 1038-2000 standard, c was clipped into a standard shape. The oxygen barrier value of Si-PC/GR nanocomposite membrane was tested on a permeability testing instrument (VAC-VBS, Labthink Ltd., Jinan, China). The average value was calculated after three tests.

### 2.9. Hydrophobicity

The water contact angle of Si-PC/GR nanocomposite membrane surface was measured on a contact angle tester (Theta, Biolin Scientific Ltd., Espoo, Finland). The average value was calculated after three tests.

## 3. Results and Discussions

### 3.1. Chemical Structures of Si-PC/GR Nanocomposites

**Figure 1:**
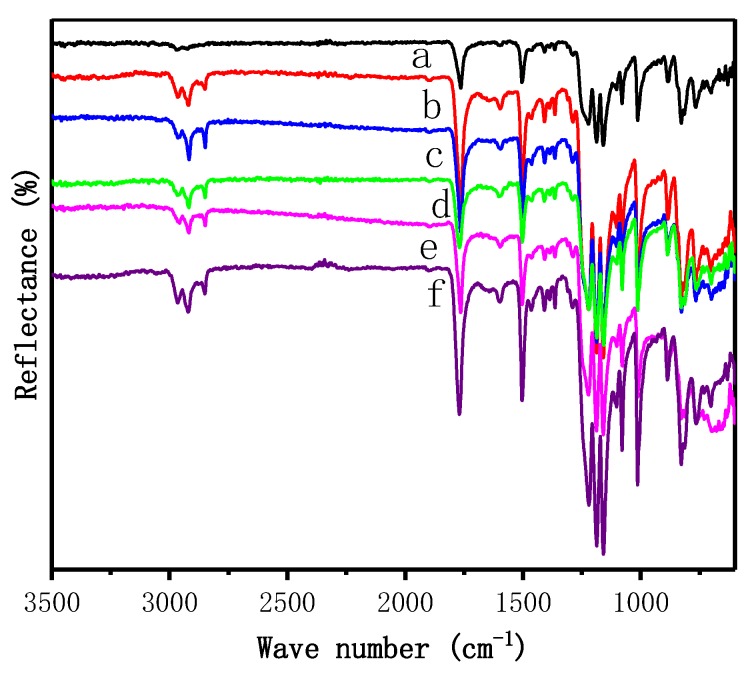
FTIR spectrograms of pure Si-PC (**a**), Si-PC/0.5%GR (**b**), Si-PC/1.0%GR (**c**), Si-PC/1.5%GR (**d**), Si-PC/2.0%GR (**e**) and Si-PC/2.5%GR (**f**).

Figure 1 shows the FTIR spectrograms of Si-PC (a), Si-PC/0.5%GR (b), Si-PC/1.0%GR (c), Si-PC/1.5%GR (d), Si-PC/2.0%GR (e) and Si-PC/2.5%GR (f). As curve a in Figure 1 shows, the absorption peaks appearing at 1220 cm^−1^ corresponds to ν C–O (ν is represent stretching vibration). The signal at 1505 cm^−1^ and 1764 cm^−1^ were corresponding to ν C=C and ν C=O. There is a weak absorption at 2950 cm^−1^ in the curve a, which is corresponding to ν C–H. These functional groups are the main groups in the Si-PC copolymer. Comparing with the curve a, the signal appeared at 2950–2800 cm^−1^, 1764 cm^−1^, 1505 cm^−1^ and 1220 cm^−1^ in the curves b–f, which were corresponding to ν C–H, ν C=O, ν C=C and ν C–O, respectively, had enhanced significantly. This was attributed to the characteristic groups in the surface or inside of graphene layers. Graphene has been oxidized slightly, and thus there are some C=Os or C–Os on the surface or inside of graphene layers. C=O and C–O—these oxygen-containing functional groups improved the compatibility between Si-PC copolymer and graphene layers, and thus further enhanced the performance of Si-PC/GR nanocomposite.

### 3.2. Morphology Analysis

**Figure 2:**
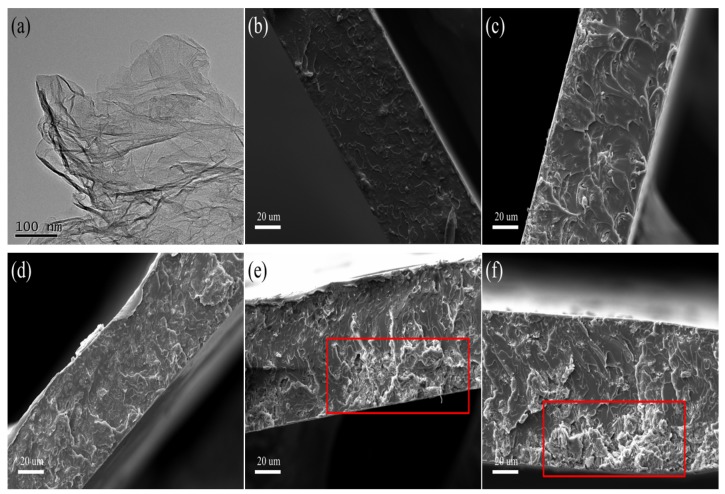
TEM image of graphene nanoparticle (**a**). SEM images of Si-PC/0.5%GR (**b**), Si-PC/1.0%GR (**c**), Si-PC/1.5%GR (**d**), Si-PC/2.0%GR (**e**) and Si-PC/2.5%GR (**f**).

Figure 2a is the TEM image of graphene stacked layers. With folding and wrinkling, graphene stacked layers exhibited a large lateral dimension. For better observing the cross-sectional morphology of Si-PC/GR nanocomposite, Figure 2b–f are the SEM images of Si-PC/0.5%GR, Si-PC/1.0%GR, Si-PC/1.5%GR, Si-PC/2.0%GR and Si-PC/2.5%GR, respectively. The fracture surface of Si-PC/0.5%GR was smooth compared with the others. With increasing addition of graphene, the roughness of the Si-PC/GR nanocomposite’s fracture surface also increased. By observing the fracture surfaces of Si-PC/0.5%GR, Si-PC/1.0%GR and Si-PC/1.5%GR, it showed that the graphene had a fine dispersion in the Si-PC, without aggregation. When the additive amount of graphene was increased to 2.0% and 2.5%, the aggregation of graphene in the Si-PC matrix was observed on the fracture surface as the red box shown in the Figure 2e,f. It was attributed to the van der Waals Force between graphene layers to result in the aggregation of graphene in the Si-PC matrix. These images illustrated that graphene with a low additive amount had dispersed well, and thus improved the performance of the Si-PC/GR nanocomposite.

### 3.3. Thermostability

**Figure 3:**
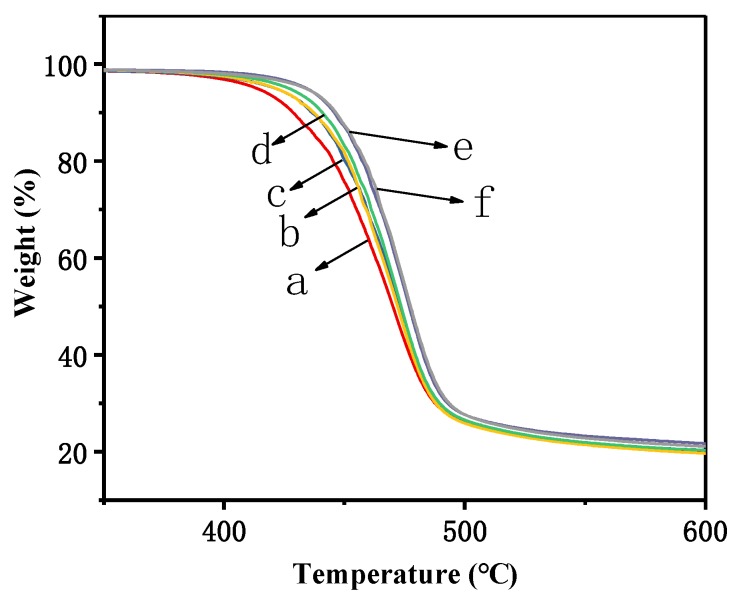
TG thermal decomposition curves of pure Si-PC (**a**), Si-PC/0.5%GR (**b**), Si-PC/1.0%GR (**c**), Si-PC/1.5%GR (**d**), Si-PC/2.0%GR (**e**) and Si-PC/2.5%GR (**f**).

Figure 3 shows the thermal decomposition curves of Si-PC (a), Si-PC/0.5%GR (b), Si-PC/1.0%GR (c), Si-PC/1.5%GR (d), Si-PC/2.0%GR (e) and Si-PC/2.5%GR (f). At 5% weight loss, the pyrolysis temperature of Si-PC was 413.7 °C. With increasing addition of graphene, the temperature at 5% weight loss of Si-PC/GR nanocomposite also increased. This temperature of Si-PC/2.5%GR nanocomposite was 434.3 °C; that was 20.6 °C higher than Si-PC. The thermostability of Si-PC/GR nanocomposite was improved slightly due to the incorporation of graphene. The thermostability of a nanocomposite is influenced by a variety of factors. This was perhaps due to the delayed release of some volatile degradation products, known as the “curved channel” effect [22]. It may be due to that the graphene had a homodispersion in the matrix, and thus there was a fine interfacial between the graphene and Si-PC matrix and mechanical interlocking structure resulting from the folding and crimping morphology of graphene, as shown in the Figure 2a [23]. Graphene with a poor dispersion in the polymer matrix also may improve the thermostability of nanocomposite due to the fact that graphene has an outstanding thermostability. However, graphene with a fine dispersion had a higher degree of enhancement in improving the thermostability of nanocomposite. Therefore, as the additive amount of graphene was over 1.5%, the pyrolysis temperature of nanocomposite was improved only slightly. The thermal stability of Si-PC/GR nanocomposite was improved only slightly, and the reason may be that graphene has high thermal conductivity, which makes it difficult for graphene to slow down the heat transfer [24].

### 3.4. Mechanical Properties

**Figure 4:**
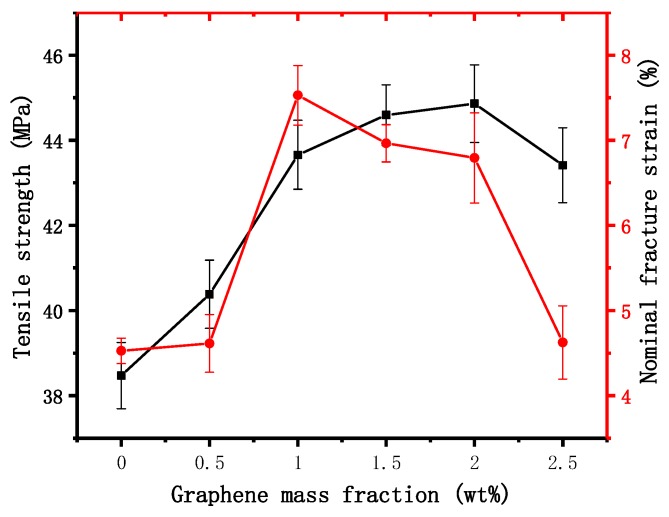
The mechanical properties of Si-PC copolymer and Si-PC/GR nanocomposites.

As shown in Figure 4, the tensile strength of Si-PC copolymer was 38.47 MPa. With increasing the additive amount of graphene to 2.0 wt%, the tensile strength of Si-PC/GR nanocomposite increased to 44.86 MPa gradually which was 6.39 MPa more than Si-PC. The tensile strength of Si-PC/2.5%GR nanocomposite decreased to 43.41 MPa. As for nominal fracture strain, the Si-PC copolymer was 4.53% and Si-PC/1.0%GR nanocomposite was 7.53%; that was maximum value in this series. As the additive amount of graphene went over 1.0%, the nominal fracture strain of Si-PC/GR nanocomposite decreased. The result illustrated that blending with graphene into the Si-PC copolymer enhanced the mechanical properties of Si-PC/GR nanocomposite in a low addition. This was due to the homodispersion of low amount of graphene in the Si-PC copolymer. The fine interfacial bonding between graphene and Si-PC contributed to reinforcing the composite. With an increasing additive amount of graphene, the mechanical properties of Si-PC/GR nanocomposite deteriorated due to the van der Waals force between layers of graphene, resulting in the aggregation of graphene causing stress concentration in the Si-PC copolymer matrix.

### 3.5. Oxygen Barrier Property

Table 1 shows the oxygen permeability values of Si-PC, Si-PC/0.5%GR, Si-PC/1.0%GR, Si-PC/1.5%GR, Si-PC/2.0%GR and Si-PC/2.5%GR nanocomposites. The oxygen permeability value of Si-PC was 213.4 cm^3^/m^2^ 24 h 0.1 MPa. As the additive amount of graphene was increased to 1.5 wt% gradually, the oxygen permeability value of Si-PC/GR nanocomposites decreased to 160.2 cm^3^/m^2^ 24 h 0.1 MPa gradually, which was 53.2 cm^3^/m^2^ 24 h 0.1 MPa less than Si-PC. The oxygen barrier property of Si-PC/GR was improved. The flawless graphene is impenetrable to all gas molecules. The improvement in the oxygen barrier property of Si-PC/GR nanocomposites was due to the homodispersion of graphene in Si-PC matrix. As the addition of graphene was over 2.0%, the oxygen permeability value of Si-PC/GR nanocomposite increased. The oxygen permeability value of Si-PC/2.5%GR nanocomposite was 231.5 cm^3^/m^2^ 24 h 0.1 MPa. This may have been due to the excess amount of graphene in the Si-PC matrix, which would have caused the aggregation of graphene, as in the SEM image shown in Figure 2e,f.

### 3.6. Hydrophobicity

**Figure 5:**
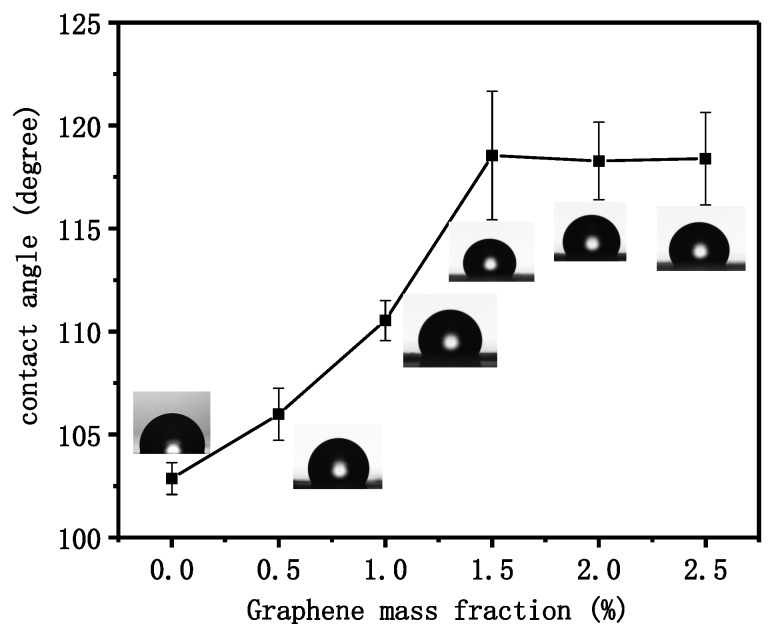
The contact angle of Si-PC copolymer and Si-PC/GR nanocomposites.

The hydrophobicity of Si-PC and of each Si-PC/GR nanocomposite was measured and the data are shown in Figure 5. The contact angle of Si-PC copolymer was 102.9°. The hydrophobicity of Si-PC/1.5%GR nanocomposite was increased to 118.5°, which was 15.7° higher than Si-PC copolymer. Graphene has a high aspect ratio and excellent hydrophobicity. Due to the incorporation of graphene, the Si-PC/GR nanocomposite had better hydrophobicity than Si-PC copolymer. As the additive amount of graphene was over 1.5%, the contact angle of Si-PC/GR nanocomposite membrane changed a little and was stable at around 118°. It may be that the contact angle 118° is close to the hydrophobicity of graphene; thus, increasing the addition of graphene cannot make much difference. The result indicated that blending with graphene had improved the hydrophobicity of Si-PC/GR nanocomposite to meet the requirement for applications in high-tech fields.

## 4. Conclusions

Upon blending graphene and Si-PC copolymer to form nanocomposite, significant changes took place. With increasing addition of graphene, the thermostability of Si-PC/GR nanocomposite was improved. At the 5% weight loss, the pyrolytic temperature of Si-PC/2.5%GR nanocomposite was 434.3 °C; that was 20.6 °C higher than Si-PC. The oxygen barrier value of Si-PC/1.5%GR nanocomposites decreased to 160.2 cm^3^/m^2^ 24 h 0.1 MPa gradually, which was 53.2 less than Si-PC. Combining the morphology and structure analysis, the homodispersion of graphene in Si-PC matrix and fine interfacial bonding between graphene and Si-PC could have contributed to improving the mechanical properties and barrier property of each Si-PC/GR nanocomposites. Each performance parameter has a variety of influencing factors and a different variation law. As the additive amount of graphene was in excess, aggregation of graphene occurred, and caused that deterioration of mechanical and barrier properties. Such aggregation had no effect on thermostability property, because graphene with a poor dispersion in the polymer matrix can still improve the thermostability of the nanocomposite due to graphene having an outstanding thermostability. The Si-PC/1.5%GR nanocomposite exhibits preferable properties, so could potentially be used as a high-performance engineering plastic to apply to high-end fields.

## Figures and Tables

**Table 1:** The oxygen permeability values of pure Si-PC, Si-PC/0.5%GR, Si-PC/1.0%GR, Si-PC/1.5%GR, Si-PC/2.0%GR and Si-PC/2.5%GR nanocomposites’ membranes.

Graphene Mass Fraction (wt%)	Oxygen Permeability (cm^3^/m^2^ 24 h 0.1 MPa)
0.0	213.4 ± 3.6
0.5	208.1 ± 2.1
1.0	205.2 ± 1.0
1.5	160.2 ± 5.5
2.0	203.2 ± 3.2
2.5	231.5 ± 2.2
